# Leaf Removal at Véraison and Foliar K^+^ Application to Beibinghong Vines Improved Berry Quality under Cold-Climate Conditions

**DOI:** 10.3390/plants11182361

**Published:** 2022-09-09

**Authors:** Zhao Le, Wei Zheng, Mengde Dong, Ming Cai, Gastón Gutiérrez-Gamboa, Baoshan Sun

**Affiliations:** 1 Faculty of Functional Food and Wine, Shenyang Pharmaceutical University, Shenyang 110016, China; 2 Service Centre for Important Industry Development of Huanren Manchu Autonomous County, Benxi 117201, China; 3 Escuela de Agronomía, Facultad de Ciencias, Ingeniería y Tecnología, Universidad Mayor, Temuco 4780000, Chile

**Keywords:** cold-climate viticulture, cold-resistant variety, grape, interspecific hybrid variety, titratable acidity, *Vitis amurensis* Rupr

## Abstract

(1) Background: Beibinghong is a grapevine variety that is well distributed in Northeastern China due to its adaptation to extreme cold conditions and vine diseases. Nonetheless, Beibinghong wines are extremely acidic and rich in phenolic compounds. The aim of this research was to study the effects of leaf removal at véraison and foliar K^+^ applications on Beibinghong vines to reduce the acidity and increase their polyphenol content. (2) Methods: Beibinghong berries were harvested when they reached close to 20 °Brix, and the physicochemical parameters were determined. (3) Results: Leaf removal at véraison plus K^+^ foliar applications to Beibinghong vines decreased the titratable acidity and increased the total phenolic and phenolic acid contents compared with the control. Moreover, the titratable acidity in the Beibinghong berries was negatively related to their total contents of phenols, proanthocyanidins, and anthocyanins. (4) Conclusions: Leaf removal at véraison performed with foliar K^+^ applications to vines could be an interesting alternative for Beibinghong production under cold-climate viticulture because it allows for a decrease in the acidity and an increase in the phenolic content of the berries, without incurring the risk of sunburn.

## 1. Introduction

Beibinghong is an interspecific hybrid variety from Zuoyouhong × Zuojia 84-26-53. It holds mostly genes from *Vitis amurensis,* and to a lesser extent *V. vinifera,* in its pedigree, and it is used to produce red ice wines [1]. *V. amurensis* is a perennial wild grapevine species that originated in East Asia and is mainly distributed in Korea, Japan, and China, and in this last country, in the Yellow River and Songhua River basins [2,3]. Northeastern China has extremely cold winters, and *V*. *vinifera* L. vines cannot survive under these conditions, even if they are protected from the cold by burying. *V*. *amurensis* is one of the most cold-resistant species in the *Vitis* genus, the branches and roots of which are capable of surviving at −40 and −16 °C, respectively, which gives it the ability to adapt to the cold conditions of Northeastern China [4]. In addition, this *Vitis* species is also highly resistant to different vine diseases, such as white rot, grape black pox, grapevine anthracnose, grape bitter rot, and downy mildew [1,4].

Beibinghong wines are rich in a wide range of secondary metabolites, and mainly polyphenols [1,5], but they are extremely acidic [1,2,5,6,7,8] because the grapes do not reach the optimal technological maturity. This has led winemakers to produce sweet red wines by adding sugar, which is hardly acceptable to most consumers. Most of the research performed to decrease the acidity in Beibinghong wines has been developed from the winemaking point of view, and scarce studies have been reported in viticulture [9]. Source–sink relationships alter the development and quality of the berries, in which source organs transport assimilates to other vegetal structures for the nutritional and reproductive vine development. Studies have shown that, in a single-canopy trellis system, values from 6 to 12 cm^2^ g^−1^ are necessary to obtain fruit with an optimum harvest maturity [10,11]. Despite this, viticulturists should also consider the species, variety, leaf area per vine or m^2^, vine level of carbon and balance translocation, leaf photosynthetic activity, carbohydrate reserves, among other variables, to reach an optimal ripeness [12,13,14]. To the best of our knowledge, there is no available information that addresses the leaf-to-fruit ratios necessary to reach the optimal technological maturity in *V*. *amurensis* varieties.

Leaf removal is a practice that is usually performed in viticulture at flowering, fruit set, or véraison to enhance the berry quality. Leaf removal at fruit set significantly reduced the malic acid content and enhanced the color intensity of Grenache wines [15], whereas leaf removal to cold-climate interspecific hybrid grapes at véraison reduced the titratable acidity, and particularly the malic acid concentration [16]. Post-véraison defoliation may decrease the berry total soluble solids at harvest and wine alcoholic content [17]. The leaf-removal effectiveness is also affected by the degree of defoliation. Cataldo et al. [18] showed that leaf removal to eight leaves at fruit set led to a slow berry ripeness, whereas the remotion of four leaves at the same phenological stage increased the sugar and anthocyanin contents in Cabernet Sauvignon grapes. Leaf removal in different positions not only improves the berry microclimate, increasing the total phenols, tartaric acid esters, anthocyanins, and flavanols in the berry skin, but also enhances bunch ventilation, reducing the grapevine-disease pressure [19].

Potassium ion (K^+^) is the most abundant cation found in grape berries, and it plays a key role in enzymatic activation and assimilate transport, controlling plant–water relationships [20,21]. Studies have shown that berries have high requirements for K^+^ upon ripening, and K additions to the soil results in more color and ripening [22]. However, the leaf ontogeny from the canopy and alterations to the source–sink balance may influence the K^+^ deficiency of grape berries.

Grape acidity results from the ratio between the free organic acids, such as malic and tartaric acids, and organic acids neutralized by K^+^ [23]. At the same, K^+^ is involved in the conversion and transport of sugars to the berries through the phloem, improving their sugar-to-acid ratio. Delgado et al. [24] showed that supplying K^+^ to vines increased the color and polyphenol content of berries by the stimulation of vine photosynthesis. Some studies have shown that the K^+^ content in vine leaf petioles were more correlated with the K^+^ content and pH in the juice than in other parts of the plant [25,26]. In addition, K^+^ absorption after soil fertilization may be affected by its interaction with other minerals in the soil [21,23], and vine roots lose their activity in the post-véraison stages. Under these conditions, foliar K^+^ applications to vines could be an interesting option to decrease the acidity in Beibinghong grape berries.

To the best of our knowledge, there are no published reports that evaluate the leaf removal of and/or foliar applications to Beibinghong grapevine varieties. Therefore, the goal of this research was to evaluate the effects of leaf removal at véraison and foliar K^+^ applications to Beibinghong vines to reduce the acidity and increase their polyphenol content in the grape berries in a preliminary study.

## 2. Results

### 2.1. Berry Weight, Physicochemical Parameters, and Phenolic Content

Table 1 shows the effect of leaf removal at véraison, the foliar potassium (K^+^) application, and their combination, to Beibinghong vines on the weight, physicochemical parameters, and phenolic compounds of the berries. The results showed that leaf removal at véraison induced a higher berry weight than the leaf removal plus K^+^ foliar application, whereas this treatment induced lower titratable acidity and higher contents of total phenols and total phenolic acids than the control. Individually, leaf removal and K^+^ foliar application treatments to vines also induced a higher total phenolic acid content in the berries than the control. The pH, total soluble solids, total proanthocyanidins, total flavonoids, and total anthocyanins were not affected by the treatments.

### 2.2. Principal Component Analysis (PCA)

**Figure 1:**
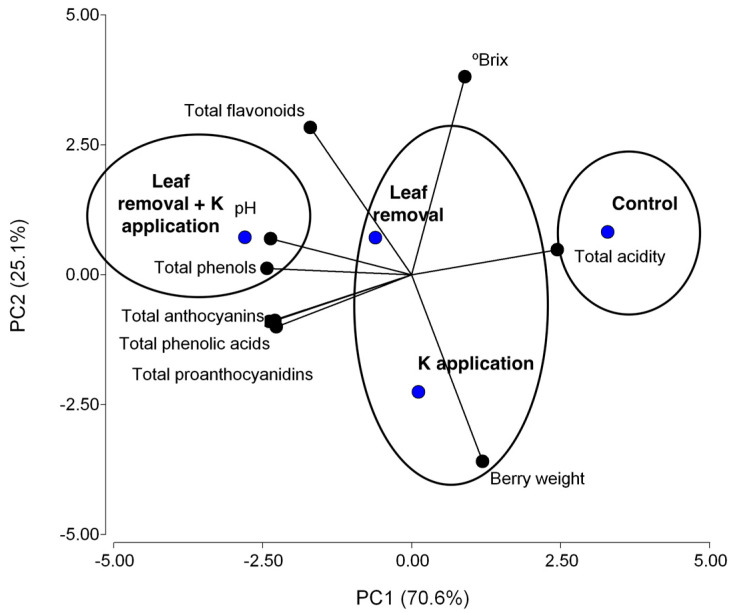
Principal component analysis (PCA) performed with berry parameters of Beibinghong vines subjected to different treatments: (i) leaf removal at véraison, (ii) potassium (K^+^) foliar application, and (iii) leaf removal plus K^+^ foliar application, as well as control. °Brix: total soluble solids. Total acidity: titratable acidity.

To classify the different samples, principal component analysis (PCA) was performed using the berry parameter data from the control and treated Beibinghong vines with different treatments: (i) leaf removal at véraison, (ii) K^+^ foliar application, and (iii) leaf removal plus K^+^ foliar application, as well as the control (Figure 1). PC1 and PC2 explained 70.6 and 25.1% of the total variance, respectively, representing 95.7% of all the variance. PC1 was strongly correlated (r^2^ > 0.9) to the pH (−), titratable acidity (+), total phenols (−), total phenolic acids (−), total proanthocyanidins (−), and total anthocyanins (−), whereas PC2 was strongly correlated to the berry weight (−) and total soluble solids (+). Leaf removal plus K^+^ foliar application was located at the opposite side of the control on PC1, whereas the leaf removal and K^+^ foliar application treatments were placed in the middle side on PC1.

### 2.3. Correlation Matrix

Table 2 shows the matrix of the correlations between nine berry parameters of Beibinghong vines subjected to different treatments: (i) leaf removal at véraison, (ii) K^+^ foliar application, and (iii) leaf removal plus K^+^ foliar application, as well as the control. The titratable acidity had negative and significant correlations with the total phenols (−0.98; *p*-value < 0.05), total proanthocyanidins (−0.96; *p*-value < 0.05), and total anthocyanins (−0.98; *p*-value < 0.01), indicating that the berries with high titratable acidity presented low contents of total phenols, proanthocyanidins, and anthocyanins. The total phenols had positive and significant correlations with the total proanthocyanidins (0.95; *p*-value < 0.05) and total anthocyanins (0.97; *p*-value < 0.05), indicating that the berries with high total phenol content also contained high contents of total proanthocyanidins and anthocyanins. The total proanthocyanidins had a positive and significant correlation with the total anthocyanins (0.98; *p*-value < 0.05), indicating that the berries with high contents of proanthocyanidins also presented high contents of anthocyanin.

## 3. Discussion

These results are the first to expose the effects of viticultural practices on *Vitis amurensis* varieties, and to provide certain interesting relationships among the berry morpho-physicochemical parameters on the Beibinghong variety. The results showed that potassium (K^+^) foliar applications to vines induced higher berry weights compared with leaf removal plus K^+^ application treatment, but not when it was compared with the control (Table 1). Some authors reported that K^+^ foliar applications to vines increased the chlorophyll content and promoted an increase in the photosynthetic products, leading to an increase in the berry weight [22,27]. However, leaf removal involves removing leaves from the basal portion of the shoots, reducing the leaf photosynthetic area, and increasing the berry sunlight exposure and air circulation [28], which could induce a decrease in the berry weight.

Based on the results, leaf removal plus K^+^ applications to the vines induced lower titratable acidity in the berries than the control (Table 1). Berries exposed to sun after leaf removal at véraison may increase their temperature, which results in the degradation of organic acids, and mainly malic acid. Sweetman et al. [29] reported that heating berries at the véraison and ripening stages reduced the malate content, which is consistent with the effects typically seen in warm vintages. K^+^ applications to vines at the post-véraison stage induced a high K^+^ mobility towards developing organs, causing the K^+^ to move from the mature leaves to the berries, which, in turn, increases the K^+^ concentration in the vacuole, generating K^+^ hydrogen tartrate, which leads to a reduction in the acidity [30]. Similar results were reported by Riesterer-Loper et al. [16], who showed that leaf removal performed at two weeks after fruit set in cold-climate interspecific hybrids increased the irradiance and temperature of the berries, reducing their titratable acidity, and particularly the malic acid content. The total phenolic and phenolic acid contents were also increased after the application of the combination of leaf removal plus K^+^ foliar applications to the vines (Table 1). Leaf removal before véraison may increase the light exposure and berry temperature, which are important factors that improve the fruit composition, color development, and phenolic compounds in the berries. Previous research trials have suggested that an increase in photosynthesis occurs when leaves are removed, and this may compensate for the reduced leaf area [31,32]. Moreover, foliar K^+^ applications to vines not only facilitate the vine growth, but also the sap flow through the phloem and xylem [20], probably improving the phenolic accumulation in the whole grape berry. Contrary to this, there were no statistical differences for the total proanthocyanidins, total flavonoids, and total anthocyanins among the treatments. Based on this, it is possible that, under cold conditions, viticultural practices such as leaf removal plus K^+^ foliar applications performed together in the vineyard could be a good strategy to decrease the high-acidity characteristic of Beibinghong berries, also improving their phenolic content.

A matrix of correlations showed that the titratable acidity in Beibinghong was negatively related to the total contents of phenols, proanthocyanidins, and anthocyanins in the berries (Table 2). In contrast, soluble solids are usually related to the anthocyanin content in most of the *V*. *vinifera* grapevine varieties [33]. Based on this, it is possible to suggest that the phenolic maturity is inversely related to the titratable acidity in Beibinghong grapes. Nevertheless, at the same level of technical maturity or soluble solids, a lower concentration of titratable acidity might be a good indicator of the phenolic maturity in this variety. Therefore, it is possible that, for these reasons, the viticultural practices only affected the acid profile and not the soluble solids content in the Beibinghong berries. With respect to the phenolic compounds, anthocyanins show pH-dependent structural isoforms in acidic and neutral solutions, but they degrade in alkaline conditions [34,35]. In the acidic vacuole, the color of anthocyanins shifts from red to blue as the pH increases [34]. In addition, it has been suggested that protocatechuic acid could be an anthocyanin-degradation product [34].

Growers in warmer locations limit leaf removal on one side of the vine canopy due to the risk of sunburn. Under Northern Chinese conditions, which are cool and sunny conditions, the partial defoliating on the cooler side may be considered to improve the berry quality. However, the risk of sunburn is high in most of the Mediterranean viticultural zones, but it is less of a concern for many regions in Northern China, where cold and overcast conditions are common. Because of the overcast conditions, the air and berry temperatures do not usually reach high enough levels of radiation for a sufficient amount of time to cause heat damage.

## 4. Conclusions

Leaf removal at véraison performed with potassium (K^+^) foliar applications to Beibinghong vines induced lower titratable acidity in the berries and higher total phenolic and phenolic acid contents than the control. Based on PCA, the control was placed at the opposite site of the plot compared with the leaf removal plus K^+^ foliar application treatment. A matrix of correlations showed that the titratable acidity in the Beibinghong was negatively related to the total contents of phenols, proanthocyanidins, and anthocyanins in the berries. Therefore, leaf removal at véraison performed together with foliar K^+^ applications to Beibinghong vines could be an interesting alternative to decrease the characteristic acidity of their grapes, even improving their phenolic compound content.

## 5. Materials and Methods

### 5.1. Plant Material

The study was conducted in 2021 in a vineyard belonging to Shenyang Pharmaceutical University (South Campus, 41.466° N, 123.710° E, Benxi, Liaoning, China), where a temperate monsoon climate predominates. Information about the climate of the plot is shown in Table 3. Beibinghong vines grafted onto Beta (*Vitis labrusca* × *V*. *riparia*) were planted in 2019, spaced at a distance of 1.0 × 2.5 m in a north–south orientation, formed in bilateral cordon, trained on a vertical-shoot-position trellis system, and spur pruned to two buds.

### 5.2. Treatments

**Figure 2:**
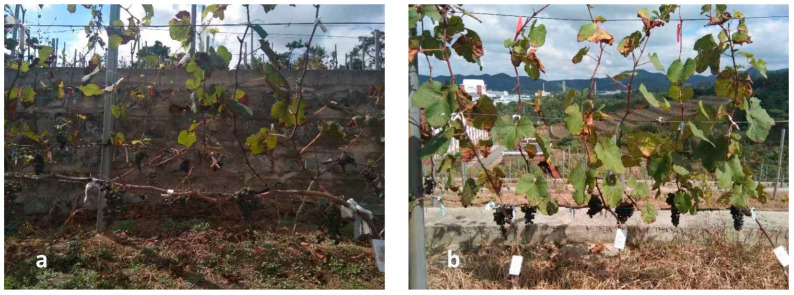
Beibinghong vines subjected to leaf removal at véraison: (**a**) compared with untreated vines or (**b**) the control.

Twelve replicates by treatment were arranged in a complete randomized block design in the vineyard, considering one healthy vine. Three treatments were performed in the vineyard, as well as the control: (i) leaf removal at véraison, (ii) foliar potassium (K^+^) application to the vines, and (iii) the combination of leaf removal and K^+^ application. The control consisted of the application of an aqueous solution, containing only Tween-80, at a dosage of 1.0%. Leaf removal was performed at véraison, removing the basal 3–4 leaves, including the basal lateral shoots (Figure 2). K^+^ foliar application was performed using a monopotassium phosphate (KH_2_PO_4_) fertilizer with a concentration of 3 g L^−1^, which was prepared with 1.0% of Tween-80 to improve the wetting area. The K^+^ solution was applied every 10 days from véraison to harvest, spraying 100 mL on both sides of the trellis, accounting for 200 mL of solution per vine. The K^+^ was applied to the vines during morning, when the stomata are open, avoiding wind and precipitation. In addition, one cluster was kept on each shoot in all treatments and the control to minimize the differences in the leaf area-to-fruit ratio in the performed analysis.

### 5.3. Harvest and Berry Determinations

Grapes were monitored to decide their optimum technological maturity to be harvested, considering the content of soluble solids close to 20 °Brix that occurred on 19 September 2021. Grapes from each replicate and treatment were harvested, placed in boxes with ice packs, and were quickly transported to the laboratory of Shenyang Pharmaceutical University. A sampling of 200 berries was taken randomly from each replicate and treatment for the subsequent determination of the berry weight, total soluble solids, pH, and titratable acidity, which were analyzed based on the OIV procedures [36]. The rest of the collected grapes were stored in a freezer at −20 °C for further analyses of the phenolic compounds.

### 5.4. Determination of Berry Polyphenols

A pretreatment to the frozen grapes was performed, in which 40 g of the frozen berries was weighed, 5 mL of water was added, and they were ground into a homogenous mass. Then, 5 g of the sample was put into a centrifuge tube, and 40 mL of 60% of analytical-grade ethanol was added. The mixture was then subjected to sonication at 50 °C for 60 min for the polyphenol extraction. Subsequently, the samples were centrifuged at 3500 r min^−1^ for 10 min, and the supernatant was taken for the analyses of the polyphenols. The total phenols, total proanthocyanidins, total flavonoids, total phenolic acids, and total anthocyanins were determined. The total phenols were determined by the Folin–Ciocalteau method, as described by Kupe et al. [37]. The total proanthocyanidins were determined by the vanillin–hydrochloric acid method, according to the report published by Mitsunaga et al. [38]. The total flavonoids were determined by the method described by Zhishen et al. [39]. The total phenolic acids were analyzed by the ferric chloride–potassium ferricyanide method, as described by Feduraev et al. [40], whereas the total anthocyanins were determined spectrophotometrically at 520 nm by the method exposed by Iland et al. [41].

### 5.5. Statistical Analysis

Statistical analysis was performed using Statgraphics Centurion XVI.I (Virginia, U.S.A), and all data were subjected to a Shapiro–Wilk normality test, Levene chi-square test, followed by a one-way ANOVA. The significance of the differences was determined by Duncan’s test (*p* ≤ 0.05). Principal component analysis (PCA) was performed to determine the relationships among the variables to classify the treatments based on the acquired data, using InfoStat (www.infostat.com.ar) (accessed on 15 August 2022).

## Figures and Tables

**Table 1:** Effects of leaf removal at véraison, foliar potassium (K^+^) foliar applications, and their combination (leaf removal plus K^+^ foliar applications) to vines on weight, physicochemical parameters, and phenolic compounds of Beibinghong berries.

	Control	Leaf Removal	K^+^ Application	Leaf Removal + K^+^ Application
Berry weight (g)	59.36 ± 10.39 ab	57.79 ± 5.89 ab	63.62 ± 6.84 b	55.34 ± 9.01 a
Total soluble solids (°Brix)	20.48 ± 1.42 a	20.16 ± 0.92 a	19.63 ± 0.10 a	20.17 ± 1.10 a
pH	3.06 ± 0.06 a	3.12 ± 0.07 a	3.09 ± 0.07 a	3.13 ± 0.08 a
Titratable acidity (g L^−1^) *	12.88 ± 0.99 b	11.96 ± 1.53 ab	11.92 ± 0.99 ab	11.28 ± 0.80 a
Total phenols (mg g^−1^)	12.95 ± 2.38 a	14.61 ± 3.21 ab	14.49 ± 2.38 ab	16.39 ± 1.96 b
Total phenolic acids (mg g^−1^)	2.07 ± 0.59 a	3.94 ± 1.02 b	3.69 ± 0.96 b	4.06 ± 0.81 b
Total proanthocyanidins (mg g^−1^)	4.25 ± 1.27 a	4.46 ± 1.12 a	4.62 ± 1.05 a	4.83 ± 0.95 a
Total flavonoids (mg g^−1^)	9.14 ± 1.96 a	10.18 ± 2.58 a	8.84 ± 1.98 a	10.22 ± 1.40 a
Total anthocyanins (mg g^−1^)	1.02 ± 0.27 a	1.07 ± 0.29 a	1.08 ± 0.25 a	1.11 ± 0.20 a

All the parameters are given with their standard error (n = 12). For each parameter, different letters indicate significant differences between treatments (Duncan test: *p*-value ≤ 0.05). * As gL^−1^ of tartaric acid.

**Table 2:** Matrix of the correlations between berry parameters of Beibinghong vines subjected to different treatments: (i) leaf removal at véraison, (ii) potassium (K^+^) foliar application, and (iii) leaf removal + K^+^ foliar application, as well as the control (above the diagonal).

	Berry Weight	°Brix	pH	Titratable Acidity	Total Phenols	Total Phenolic Acids	Total Proanthocyanidins	Total Flavonoids	Total Anthocyanins
Berry weight	1								
°Brix	−0.64	1							
pH	−0.58	−0.23	1						
Titratable acidity	0.39	0.46	−0.91	1					
Total phenols	−0.52	−0.30	0.91	−0.98 *	1				
Total phenolic acids	−0.23	−0.58	0.92	−0.92	0.85	1			
Total proanthocyanidins	−0.27	−0.51	0.77	−0.96 *	0.95 *	0.82	1		
Total flavonoids	−0.90	0.34	0.83	−0.59	0.66	0.57	0.40	1	
Total anthocyanins	−0.29	−0.53	0.86	−0.99 **	0.97 *	0.91	0.98 *	0.49	1

Data in bold correspond to a significant relationship. In the table, ** and * indicate significance at 1 and 5% of probability, respectively, according to a *t*-test. Twelve observations underlaying the correlations.

**Table 3:** Climatic information of the experimental site provided by the Benxi Meteorological Administration during 2021 season.

	2021
Growing Season (from April to September)	
Precipitation (mm)	1097
ET_0_ (mm)	554
Minimum temperature (°C)	−2.7
Average temperature (°C)	19.6
Maximum temperature (°C)	34.3
Relative humidity (%)	0.7
Accumulated radiation (MJ m^2^)	5166.7
Warmest Month (July)	
ET_0_ (mm)	114.4
Average radiation (MJ m^2^)	17.74
Annual	
Precipitation (mm)	1342
ET_0_ (mm)	676.98

Abbreviation: ET_0_: reference evapotranspiration.

## Data Availability

Not applicable.
